# Advancing arrhythmia education through the CDIO approach: a new paradigm in nursing student training

**DOI:** 10.1186/s12912-024-02118-1

**Published:** 2024-06-25

**Authors:** Yu Chen, Heling Wen, Zheng Huang, Rui Zhang, Lei Peng

**Affiliations:** 1 Department of Cardiology, School of Medicine, Sichuan Provincial People’s Hospital, University of Electronic Science and Technology of China, Chengdu, 610072 China; 2 Department of Surgery, The Affiliated Tumor Hospital of Chengdu Medical College, Chengdu, 610021 China; 3 Institute of Nephrology, Sichuan Provincial People’s Hospital, School of Medicine, University of Electronic Science and Technology of China, Chengdu, 610072 China

**Keywords:** Arrhythmia, CDIO model, Lecture-based learning, Nurses

## Abstract

**Background:**

The accurate diagnosis and effective management of arrhythmias are crucial, with nurses playing a key role in the early detection and treatment, significantly impacting patient outcomes. Improving education on arrhythmias among nurses, especially in critical care and perioperative settings, can enhance patient safety and the quality of care.

**Methods:**

A total of 116 trainee nurses were randomly divided into two groups: one utilizing the conceive-design-implement-operate (CDIO) model and the other employing a traditional lecture-based learning (LBL) method, to undergo arrhythmia training. The studyassessed the effects of the two teaching methods and investigated the students’ attitudes toward these educational practices, with all participants completing pre- and post-course tests.

**Results:**

The CDIO model significantly enhances nursing students’ arrhythmia proficiency, yielding higher test scores and sustained improvement after 24-week compared to the traditional LBL method, alongside markedly better self-learning enthusiasm, understanding, satisfaction with the teaching approach and effectiveness, and interest in learning arrhythmia. The CDIO model in nursing arrhythmia courses boosts theoretical knowledge and application, showing potential in clinical skill enhancement.

**Conclusions:**

Our study introduces the CDIO model in nursing arrhythmia courses, with improvement in knowledge and skills, and promise for broader application.

**Supplementary Information:**

The online version contains supplementary material available at 10.1186/s12912-024-02118-1.

## Introduction

Arrhythmia encompasses any deviation from the normal cardiac rhythm, manifesting as tachycardia, bradycardia, or irregular heartbeats. This condition originates from disruptions in the cardiac electrical system, varying from benign to life-threatening. Severe arrhythmias can compromise cardiac function and elevate risks of stroke, heart failure, or sudden cardiac arrest. The diagnostic and therapeutic approach for arrhythmias typically involves electrocardiographic monitoring and may include pharmacological interventions, lifestyle modifications, or procedural treatments. The choice of therapy is contingent upon the specific arrhythmia type, its etiological factors, and the patient’s overall health condition. Crucially, arrhythmias in hospitalized patients are pivotal in determining prognosis, necessitating timely identification and management to mitigate associated risks.

Nurses often are front-line responders to cardiac emergencies such as cardiac arrests and tachycardia in hospitalized patients, playing a vital role in the timely identification and intervention of lethal arrhythmias. Their rapid and accurate interpretation skills are critical in enhancing patient outcomes, highlighting their pivotal role in acute cardiac care [1]. Recent advancements in medical science and technology have led to an increased demand for arrhythmia monitoring across all patient demographics, regardless of their specific ward or department [2]. Prompt detection and effective management of patient deterioration significantly impact patient outcomes. The issue of “failure to rescue”, a global healthcare concern, is partly due to nurses’ challenges in detecting and managing patient deterioration, particularly in identifying and handling cardiac arrhythmias [3].Furthermore, a study by Goodridge et al. found that among surgical nurses, 48% of abnormal electrocardiograms (ECGs) were not interpreted satisfactorily, potentially affecting the medical safety of surgical patients. This emphasizes the necessity for improved training and support for nurses in ECG interpretation to ensure patient safety and quality care in surgical settings [4].

Recent research underscores the imperative for ongoing education and training to equip nurses with the proficiency needed for accurate cardiac rhythm interpretation, a cornerstone of exemplary patient care and treatment outcomes [2]. Studies indicate a strong correlation between nurses’ arrhythmia training and their ability to diagnose and manage high-risk arrhythmias, especially in critical care settings such as the Coronary Care Unit (CCU), Intensive Care Unit (ICU), and Emergency Department [5–7]. In these settings, patients often present clinical instability and are under continuous non-invasive cardiac monitoring.

In contrast, nurses in surgical wards generally receive less training in arrhythmia management, which is concerning given that perioperative arrhythmias are a common and potentially severe complication in surgical patients [8]. Atrial fibrillation (AF), prevalent in 16–30% of post-cardiac and thoracic surgeries, poses serious risks, including organ hypoperfusion, pulmonary edema, and myocardial infarction. The incidence of perioperative arrhythmias in non-cardiothoracic surgeries varies between 4 and 20%, influenced by the type of surgery, patient health, and surgical stressors [9]. Notably, the incidence of arrhythmias, particularly AF, can range from 2 to 60% in cardiothoracic and esophageal surgeries [10].These findings necessitate vigilant monitoring and proactive management of arrhythmias in perioperative settings to avert severe complications. Enhancing the medical safety of perioperative surgical patients thus mandates essential arrhythmia training for nurses in surgical departments, aligning with the broader goal of optimizing patient outcomes and safety in high-risk clinical environments.

Theoretical knowledge and practical skills of arrhythmia is an essential skill for all nurses [11]. Timely identification and management of life-threatening arrhythmias by nurses can reduce mortality rates and improve patient outcomes [12]. A study shows that most practicing nurses exhibit a positive attitude towards the diagnosis of arrhythmias, but the majority demonstrate a lower level of proficiency in arrhythmia diagnosis [13]. Another study found that nursing students have a certain gap compared to practicing nurses in both theoretical knowledge and practical skills [14]. The deficiencies in arrhythmia knowledge and skills among nurses and nursing students may stem from insufficient education and training, making it crucial to provide appropriate educational opportunities to improve the competency of nursing personnel in managing arrhythmias [15, 16]. Providing training in arrhythmia management to nursing students may positively impact their future practice capabilities, establishing a foundational knowledge base that facilitates specialized training requiring a solid understanding of arrhythmias [17].

Within the realms of clinical medicine and nursing education, traditional lecture-based learning (LBL) method presents distinct advantages and drawbacks. Its primary utility lies in the efficient dissemination of comprehensive theoretical knowledge and professional insights to large student cohorts, forming an essential foundation for their educational journey. LBL method ensures a standardized method of content delivery, which is fundamental for upholding the quality and consistency of nursing education [18]. Nevertheless, the limitations of LBL method are significant. It often lacks dynamic interaction and engagement, which may lead to reduced student interest and participation. Given the practical nature of nursing, LBL method might fall short in addressing the hands-on skills and real-world applications critical for clinical practice [19, 20]. Additionally, its generalized approach may not cater to the varied students’ learning style. Research suggests that compared to more interactive and experiential teaching methodologies, LBL method might not be as effective in promoting deep learning or in fostering long-term retention of knowledge [7]. Therefore, while LBL method is valuable for knowledge transmission, its efficacy is greatly enhanced when integrated with interactive, learner-centered educational strategies.

The Conceive-Design-Implement-Operate (CDIO) educational framework is a pioneering approach that focuses on a hands-on, practical learning process [21]. This model was developed as a response to the growing need for students to be adept not only in knowledge but also in skills such as problem-solving, teamwork, and innovation [21, 22]. The CDIO framework was initially conceptualized and developed by a group of engineering educators from the Massachusetts Institute of Technology (MIT). The development of this framework was driven by the recognition that traditional education often lacked sufficient emphasis on real-world problem-solving and practical skills. This model allows students to engage in learning through the actual experience of practices, rather than through theoretical study alone. These advantages align well with the pedagogical characteristics of clinical medicine and nursing, as these disciplines are inherently practice-oriented, necessitating the cultivation of students adept in both theoretical knowledge and practical skills to serve patients and enhance healthcare quality.

In recent years, the CDIO model has gradually gained wider application within the nursing education system. Instructors utilize the CDIO model in various nursing student training programs, such as cardiovascular health behavior modification, orthopedic nursing, and the cultivation of core competencies [23–25]. Xinyang Su et al. found that the CDIO model can stimulate the independent learning and critical thinking abilities of nursing interns, promote the organic integration of theory and practice in orthopedic nursing [23]. Xinyue Dong et al. discovered that the CDIO model enhances nursing students’ health education skills, increases their perception of clinical decision-making, and optimizes their ability to conduct behavior change counseling [24]. Another study indicated that within the training of neurosurgical nurses, the CDIO model can improve students’ core competencies and general self-efficacy [25]. Currently, we are not clear on whether the new CDIO teaching model is more suitable for arrhythmia education among nursing students compared to traditional teaching methods. It remains to be explored whether this model offers advantages over the traditional LBL teaching approach in terms of both theory and practice of arrhythmia, and these questions are worth investigating to answer.

Our research hypothesizes that the CDIO approach can better achieve the objective of enhancing arrhythmia education among nursing interns. By comparing it with the traditional LBL method, this study aims to assess the effectiveness of these two distinct teaching methodologies in arrhythmia, particularly in the capability of diagnosing arrhythmias, and to investigate student attitudes towards this educational practice. To our knowledge, no studies have yet analyzed the effectiveness of the CDIO approach in the education of arrhythmias among nursing interns.

## Methods

### Study design

This is a randomized controlled trial with two groups, encompassing three arrhythmia tests and a quantitative questionnaire survey. The study was conducted at a training and research hospital during the academic year 2022–2023. The PASS 15.0 (Power Analysis and Sample Size) Software (UT, USA) was utilized for the calculation of sample size. Prior to initiating the research, we estimated the sample size using the outcomes from our preliminary study. With an alpha of 0.05 and a power of 0.9, we determined a requirement for 52 subjects in each group. Accounting for a 10% loss of samples, the adjusted number of subjects for each group was established at 58 participants per group. A total of 116 third-year nursing students were enrolled in the study during their internships in Department of Surgery. These students were randomly divided into two groups using a digital randomization method. Fifty-eight nursing students were trained using the CIDO method as the experimental group, while the other fifty-eight were taught using the traditional LBL method as the control group. This research was conducted with the approval of the Institutional Review Board and Ethics Committee of the Affiliated Tumor Hospital of Chengdu Medical College, and informed consent was obtained from each participant (Ref: 36-2-1). This study was conducted in accordance with the 2013 revision of the Declaration of Helsinki [26].

### Teaching implementation

#### The CDIO model for arrhythmia course

**Fig. 1:**
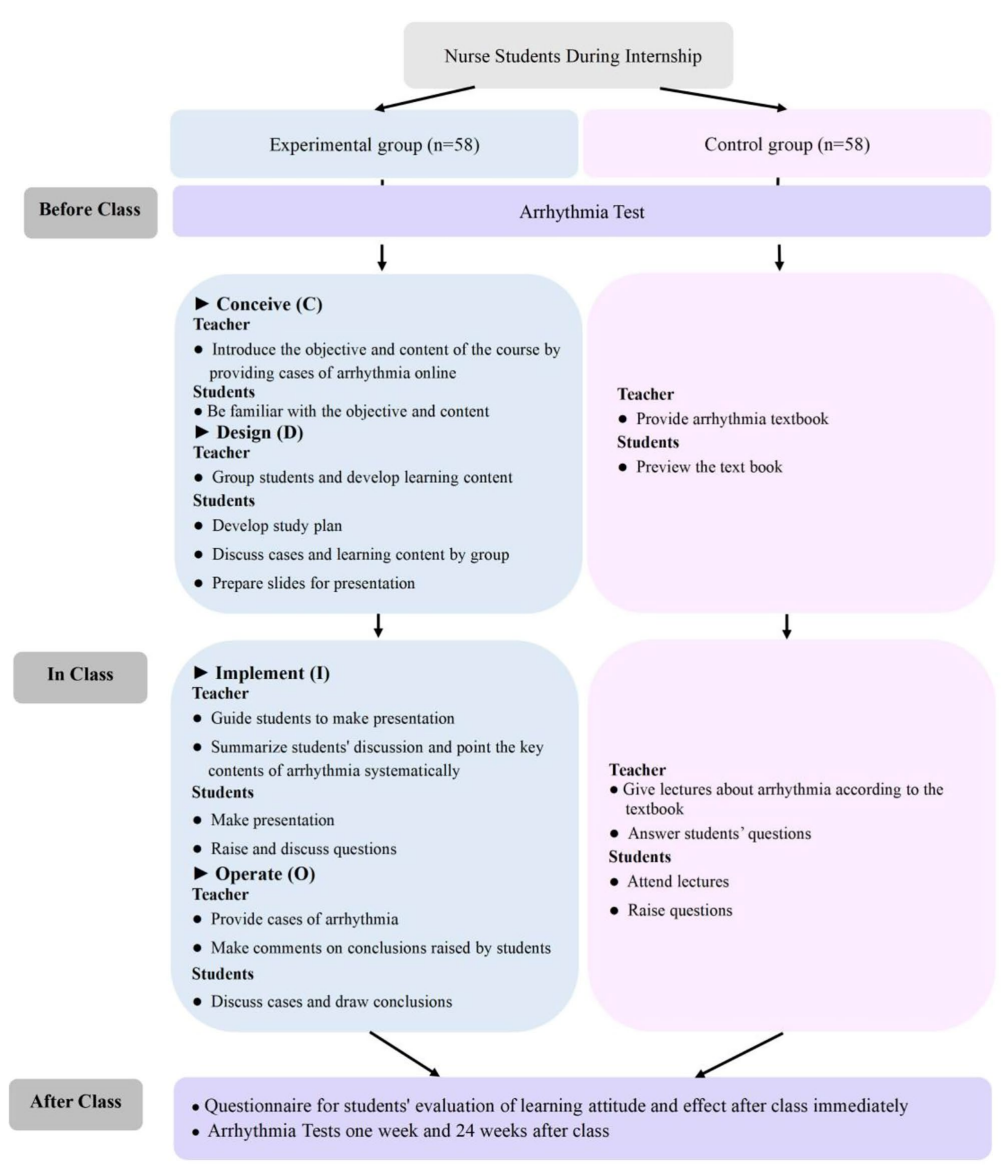
Study design. Control group: traditional LBL method; Experimental group: CDIO method

This course categorizes arrhythmias into two types: tachyarrhythmias and bradyarrhythmias, with each type of arrhythmia covered over two class periods, each lasting 45 min. The specific steps of CDIO for arrhythmia teaching are as follows (Fig. 1) :

##### Conceive (C):

Before the class, the instructor will present students with typical electrocardiogram (ECG) cases of arrhythmias, posing questions and introducing the course’s objectives and content through these cases. Questions might include: What diagnosis is currently being considered for the patient? What are the common clinical symptoms? What are the ECG findings? How does it differ from other arrhythmias? What are the treatment options? etc. After posing these questions, students are encouraged to make preliminary attempts at answering them, becoming familiar with the course content and objectives. This approach is designed to create a sense of urgency and to stimulate their enthusiasm for learning. The questions are centered around the student’s learning outcomes for arrhythmia teaching: (1) Understanding the definition and classification of arrhythmias. (2) Mastering the ECG diagnosis and differential diagnosis of arrhythmias. (3) Learning about the treatment methods for arrhythmias.

##### Design (D):

Before class, tasks with the potential to attract students are designed to stimulate their enthusiasm for learning. Teachers organize students into groups (8–10 members each, with a group leader elected to assist the teacher with educational activities and to distribute tasks within the group), design, and assign learning tasks. Starting from typical cases, problems, and assigned tasks, students engage in group discussions on cases and learning materials, formulate learning plans, and prepare slides for subsequent classroom presentations.

##### Implement (I):

In classroom teaching, teachers play a mediating role, guiding students to focus on learning tasks to facilitate their completion. Students utilize learning materials provided by the teacher and consult other relevant resources to analyze typical cases in the driving phase and answer related questions. They work in groups to summarize and organize the issues encountered during group discussions, presenting the content in slideshow format in the classroom and providing feedback to the teacher, a process that lasts 20 min. Based on the students’ group discussion learning, the instructing teacher summarizes and delivers theoretical knowledge lectures, addresses questions raised by the students, and explains difficult concepts and challenging points, a process that lasts 10 min.

##### Operate (O):

In classroom teaching, teachers use actual patient records from the hospital to guide students in reviewing knowledge. Students are encouraged to express their diagnostic and treatment opinions, which are then evaluated and corrected by the teacher. Students engage in task output through active participation and answering questions, allowing the teacher to observe the quality of their output in real-time. When a student’s logical thinking is found to be poor, their answers incomplete or incorrect, timely feedback and critique are provided to deepen their understanding of the concepts and continuously improve the quality of their output. The teacher will give a final explanation for questions with a low correctness rate and conclude the session once it is confirmed through classroom Q&A that students have a basic grasp of the knowledge points. This process lasts 15 min.

#### LBL method for ECG course

The LBL method for ECG course comprised four sessions, each lasting 45 min. Prior to the class, the teacher provided the students with an arrhythmia textbook for preview. Throughout the course, the teacher conducted a 40-minute lecture to elucidate various arrhythmias, which was followed by an approximately 5-minute Q&A session (Fig. 1).

#### Assessment of teaching effect and students’ attitudes

To assess the students’ understanding and application of knowledge, the two groups had the same examinations, including one test before course and two after course which was adopted before training, one week and 24-week after training respectively. According to the Bloom’s Taxonomy, all questions in the test were categorized into two aspects, basic theoretical knowledge (25 points) and clinical case analysis (25 points). The basic theoretical knowledge section consisted of 25 multiple-choice questions on arrhythmias, with each question worth 1 point. Similarly, the clinical case analysis section assessing students’ application of knowledge included 25 questions on arrhythmia cases, with each also worth 1 point. The format for both sections was multiple-choice, and each question had only one correct answer. The examples of questions that pertain to both basic theoretical knowledge and clinical case analysis are included in the Supplemental Materials (Figure S1-S2). The questions in the three tests were assessed to ensure consistency in difficulty levels by two different teachers. The total score was 50 points and the test time was 60 min. We calculated the total score, theoretical and application scores of each test for each student.

To assess the students’ evaluation of learning attitudes and effects, a questionnaire survey was adopted at the end of the course, including self-learning enthusiasm, study load, systematization of teaching content, understanding of teaching content, student-teacher interaction, satisfaction of teaching mode, satisfaction of teaching effect, development of self-confidence, team collaboration and interest in learning arrhythmia (Supplemental Table S1). The 5-level Likert scoring method was adopted for each question, with 5 points for very satisfied/strongly agreed, 4 points for satisfied/agreed, 3 points for neutral, 2 points for dissatisfied/disagreed and 1 point for very dissatisfied/strongly disagreed.

### Statistical analysis

Data normality was evaluated using the Shapiro–Wilk test. Data were presented as mean ± standard deviations (SDs) or median (interquartile range, IQR) values, as appropriate according to data distribution. Ages were compared with the Mann–Whitney U test. Arrhythmia test scores before training of the two groups were compared by t-test. For the analysis of the arrhythmia test scores at different time points, comparisons were analyzed using analysis of variance (ANOVA) for repeated measures. For the 5-level Likert scores of students’ evaluation of learning attitudes and effect, the Mann–Whitney U test was applied. Statistical analyses were conducted in SPSS 26.0 (SPSS Inc., Chicago, USA). All tests were two-tailed, and significance was set at *p* < 0.05.

## Results

### Baseline information of participants

A total of 116 nursing students undergoing internships were enrolled in this study and were randomly divided into two groups. All enrolled nursing students were female. The experimental group comprised 58 nursing students and utilized the CDIO method, while the control group consisted of 58 nursing students and used the LBL method. Upon comparison of factors such as age and arrhythmia test scores before the start of the curriculum, no significant differences were found between these two groups (*z* =-0.407, *p* > 0.05 for age; *t* = 0.857, *p* > 0.05 for total arrhythmia test scores; *t* = 0.105, *p* > 0.05 for theoretical arrhythmia test scores; *t* = 1.384, *p* > 0.05 for application arrhythmia test scores; Table 1).

**Table 1:** Baseline characteristics of enrolled nursing students

Characteristics	Control Group (*n* = 58)	Experimental Group (*n* = 58)	z/t	*p*
Age (Yrs)	20.0 (20.0–21.0)	20.0 (20.0–21.0)	-0.407	0.684^a^
Total arrhythmia test scores before training	21.85 ± 2.82	22.31 ± 3.03	0.857	0.393^b^
Theoretical arrhythmia test scores before training	11.91 ± 2.52	11.86 ± 2.77	0.105	0.917^b^
Application arrhythmia test scores before training	9.93 ± 1.84	10.45 ± 2.17	1.384	0.169^b^

^a^The two groups were compared using a Mann–Whitney *U* test; ^b^The two groups were compared using an independent sample *t*-test

### Arrhythmia test scores in two groups

**Fig. 2:**
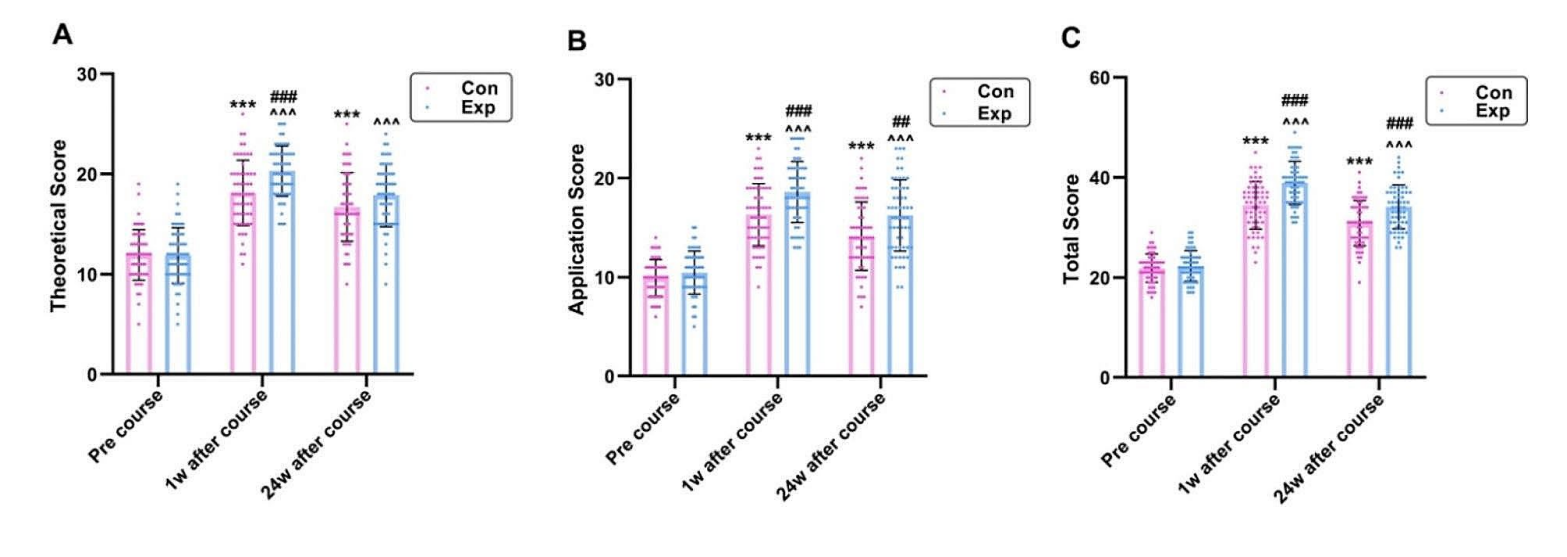
Scores of arrhythmia tests in Con (*n* = 58) and Exp (*n* = 58) groups at different time points. **A**, Theoretical Score; **B**, Application Score; **C**, Total Score. Con: control group with the traditional LBL method. Exp: experimental group with the CDIO model. Data are presented as mean ± standard deviation (SD). ***p < 0.001 vs. Pre-Course in Con group, ^^^*p* < 0.001 vs. Pre-Course in Exp group, ###*p* < 0.001 vs. scores in Con group at 1w and 24w after course ,## *p* < 0.01 vs. scores in Con group at 24w after course

The arrhythmia test scores for the two groups are presented in Fig. 2. One week prior to the commencement of the arrhythmia course, the students underwent a pre-test assessment. In the experimental group, the mean total score was 22.31 ± 3.03, with mean scores for theoretical knowledge and application being 11.86 ± 2.77 and 10.45 ± 2.17, respectively. For the control group, the corresponding mean scores were 21.85 ± 2.82 for the total, 11.91 ± 2.52 for theoretical knowledge, and 9.93 ± 1.84 for application. There were no significant differences between the experimental and control groups (*p* > 0.05), indicating that the baseline characteristics of these two groups were comparable.

There were marked increases in the experimental group’s total scores, theoretical knowledge scores, and application scores: 22.31 ± 3.03 at baseline, 38.90 ± 4.33 one week after training and 34.10 ± 4.38 twenty-four weeks post-training (*F* = 283.159, *p* < 0.001); 11.86 ± 2.77 at baseline, 20.29 ± 2.51 one week after training and 17.88 ± 3.13 twenty-four weeks post-training (*F* = 126.013, *p* < 0.001); 10.45 ± 2.17 at baseline, 18.60 ± 3.08 one week after training and 16.22 ± 3.61 twenty-four weeks post-training (*F* = 172.044, *p* < 0.001), respectively. Similarly, in the traditional group, these scores were as following: 21.85 ± 2.82 at baseline, 34.43 ± 4.76 one week after training and 30.84 ± 4.51 twenty-four weeks post-training (*F* = 163.439, *p* < 0.001); 11.91 ± 2.52 at baseline, 18.12 ± 3.26 one week after training and 16.71 ± 3.43 twenty-four weeks post-training (*F* = 69.933, *p* < 0.001); 9.93 ± 1.84 at baseline, 16.31 ± 3.15 one week after training and 14.14 ± 3.44 twenty-four weeks post-training (*F* = 102.690, *p* < 0.001), respectively.

One week after training, the experimental group’s total scores, theoretical knowledge scores, and application scores were significantly higher than those of the traditional group (38.90 ± 4.33 vs. 34.43 ± 4.76, *F* = 27.962, *p* < 0.001; 20.29 ± 2.51 vs. 18.12 ± 3.26, *F* = 16.182, *p* < 0.001; 18.60 ± 3.08 vs. 16.31 ± 3.15, *F* = 15.714, *p* < 0.001).

Twenty-four weeks post-training, the experimental group showed total scores, theoretical knowledge scores, and application scores of 34.10 ± 4.38, 17.88 ± 3.13, and 16.22 ± 3.61, respectively. Conversely, the control group exhibited scores of 30.84 ± 4.51 for total, 16.71 ± 3.43 for theoretical knowledge, and 14.14 ± 3.44 for application. Significant disparities were observed between the two groups in terms of total and application scores 24 weeks after training (*F = 15.540, p* < 0.001 and *F = 10.140*, *p* = 0.002, respectively), whereas the theoretical knowledge scores did not differ significantly. The detailed statistic and *p* value of arrhythmia test scores ware showed in Supplemental Table S2.

### Comparison of students’ attitudes

**Fig. 3:**
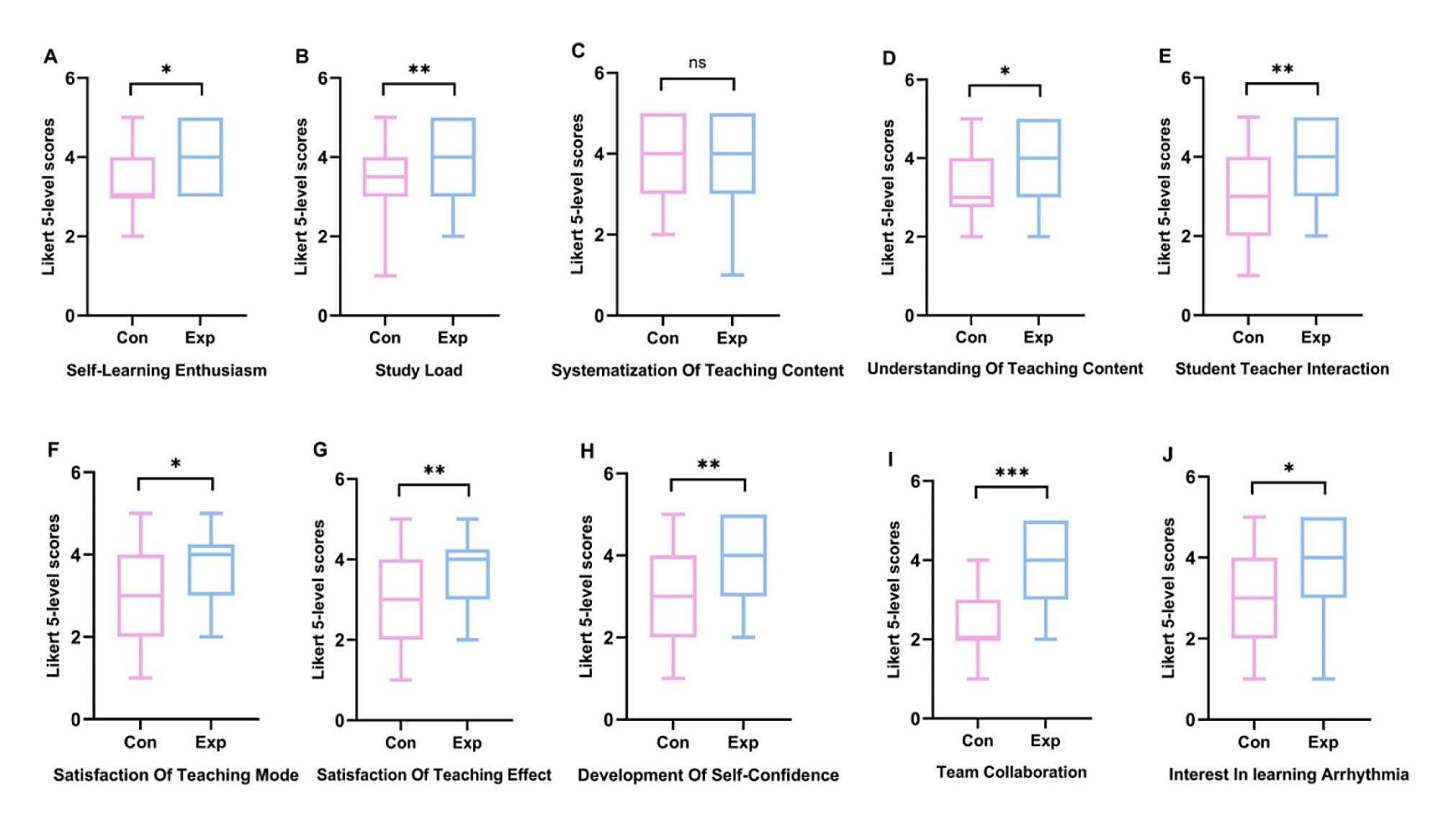
Five-level likert scores of students’ attitudes in Con (*n* = 58) and Exp (*n* = 58) groups. **A**, Self-Learning Enthusiasm; **B**, Study Load; **C**, Systematization Of Teaching Content; **D**, Understanding Of Teaching Content; **E**, Student Teacher Interaction; **F**, Satisfaction Of Teaching Mode; **G**, Satisfaction Of Teaching Effect; **H**, Development Of Self-Confidence; **I**,Team Collaboration; **J**, Interest In learning Arrhythmia. Con: control group with the traditional LBL method. Exp: experimental group with the CDIO model. ns: no significant difference, *p < 0.05 Exp vs. Con, **p < 0.01 Exp vs. Con, ***p < 0.001 Exp vs. Con

A total of 116 questionnaires were distributed, all of which were returned, yielding a recovery rate of 100%. When comparing the experimental group with the control group, significant improvements were noted in the experimental group in various aspects. These improvements included self-learning enthusiasm (*z*=-2.197, *p* = 0.028), comprehension of the teaching content (*z*=-2.566, *p* = 0.010), student-teacher interaction (*z*=-2.621, *p* = 0.009), satisfaction with the teaching mode (*z*=-2.362, *p* = 0.018), satisfaction with the teaching effectiveness (*z*=-2.696, *p* = 0.007), development of self-confidence (*z*=-3.358, *p* = 0.001), team collaboration (*z*=-7.843, *p* < 0.001), and interest in learning about arrhythmia (*z*=-2.173, *p* = 0.030). However, the organization of teaching content was found to be similar between the two groups (*z*=-1.030, *p* = 0.303). Additionally, a total of 72.4% students in the experimental group reported an increased study load (*z*=-3.392, *p* = 0.001) (Fig. 3).

## Discussion

Proficiency in managing arrhythmias is crucial for nurses as it directly affects patient safety [27, 28]. Traditional teaching methods have proven less effective in educating nurses about arrhythmias, highlighting an urgent need to explore new methods to enhance the educational outcomes for nursing students in this area [29, 30]. For the first time, we investigated the application of the CDIO model in teaching arrhythmia to nursing students. Our findings indicate that, compared to the traditional LBL method, the CDIO model significantly improves students’ theoretical knowledge and practical skills in managing arrhythmias. Additionally, student feedback suggests that the CDIO model outperforms the LBL method in terms of self-learning enthusiasm, understanding of teaching content, student-teacher interaction, students’ satisfaction of teaching mode, and more.

In nursing education, the LBL method remains the mainstream approach, but it is prone to inducing passive learning, which diminishes student engagement, interest, and motivation for independent learning [31, 32]. Given the limitations of the LBL method, it is imperative that we seek innovative teaching approaches. It has been shown that the new methods significantly elevate the caliber of nursing education by promoting active engagement and critical analysis, and also positively impact patient care [33–36]. Our study indicates that the CDIO model group participants outperformed the control group in both theoretical knowledge assessment and application capability evaluation, with a statistically significant difference after training (*F* = 12.116, *p* = 0.001 for theoretical knowledge; *F* = 23.681, *p* < 0.001 for application capability; Table S2). The CDIO teaching model integrates students into the curriculum with practical problems right from the “Conceive” phase. Moreover, during the “Operate” phase, the study of actual cases further transforms arrhythmia theoretical knowledge into practical applications. Numerous studies underscore the efficacy of the CDIO framework in nursing education, particularly its role in enhancements in the understanding and application of knowledge [23, 24, 37, 38]. A study on nursing students in orthopedic internships demonstrated that the CDIO model significantly enhances clinical competencies, analytical thinking, and self-directed learning by effectively integrating theoretical understanding with practical skills, thereby enriching problem-solving abilities and teaching effectiveness [23]. Furthermore, another research indicated that online courses utilizing the CDIO model surpassed traditional methods in theoretical knowledge and practical skill assessments, thereby bolstering health education proficiency and clinical decision-making acumen [24]. Additionally, a study on CDIO model for nursing students in respiratory and critical care medicine internships indicated that students in the CDIO group scored higher than those in the control group in both theoretical and practical exams, demonstrating effective teaching [37]. Moreover, in endocrinology nursing skill training, the CDIO model has shown advantages over traditional approaches, with students outperforming the control group in Mini-Clinical Evaluation Exercise scores, instructor evaluations, and patient satisfaction surveys [38]. Collectively, these studies highlight the CDIO model’s multifaceted applications in nursing education, proving its effectiveness in enhancing both knowledge and its application.

This improvement in teaching effectiveness may stem from the unique instructional design of the CDIO model. In our CDIO model for teaching arrhythmias, the “Conceive” phase starts with presenting typical arrhythmia cases, immersing nursing students in scenarios. Case-related questions encourage students to preview content and consult relevant literature, sparking their interest. In the “Design” phase, students actively engage in problem-solving, enhancing self-directed learning and enthusiasm. The “Implement” phase features group presentations and teacher feedback, with the teacher transitioning from “knowledge delivery” to “activity guidance.” In the “Operate” phase, in-hospital arrhythmia cases strengthen knowledge integration and practical skills.

Student feedback is an important basis for evaluating teaching methods, helping to develop more scientific course designs and teaching strategies to improve teaching effectiveness. Therefore, we observed student feedback on the application of the CDIO model in teaching arrhythmias from multiple perspectives.

We found that the CDIO model kindles students’ self-learning enthusiasm. The introduction of cases before classroom sessions requires students to be proactive in their learning process prior to classroom teaching, seeking and utilizing various resources to solve problems. The act of confronting challenges and solving problems in itself serves as an incentive, encouraging students to actively seek solutions, thereby bolstering their self-learning enthusiasm [37]. Through the practical activities during the “Operate” phase, students are able to see the direct outcomes and significance of their learning, thereby further stimulating their self-learning enthusiasm.

We found that compared to the traditional LBL method, CDIO increases students’ study load, as traditional teaching methods only require passive knowledge reception. The CDIO model necessitates active student participation in classroom activities. Additionally, they must confront immediate feedback from peers and teachers, a process that could heighten their energy expenditure. The increase in the study load has also been observed in other studies of non-traditional teaching models that transform students from passive recipients in the classroom to active participants [39, 40].

Our study found that the CDIO model group does not have an advantage over the traditional LBL group in terms of the systematization of teaching content. The CDIO model focuses on cultivating students’ ability to apply knowledge in arrhythmia, making it challenging for students to ensure a balanced and in-depth understanding across all types of arrhythmias during their learning process. The autonomous nature of student learning may lead to inconsistencies in the content and depth of learning, thereby affecting the systematic construction of the knowledge system. Furthermore, the shift of teachers from traditional knowledge transmission to guiding and collaborating in learning could also impact the systematic organization and conveyance of teaching content.

The abstract nature of arrhythmia knowledge presents a challenge for nursing students’ learning. How to enhance students’ understanding of teaching content is a crucial focal point in the reform of teaching methods [41, 42]. The CDIO model emphasizes deepening theoretical knowledge through the study of actual clinical cases. By applying abstract theories to the analysis and handling of specific cases, students can intuitively grasp the application of theory in practice. This “learning by doing” approach aids in enhancing students’ understanding of teaching content.

Our study found that increased student teacher interaction is a significant characteristic of the CDIO model. This approach transforms the classroom into a platform for student teacher interaction, fostering a more active, interactive, and personalized learning environment. Teachers facilitate student participation in discussions and assist students in recognizing their progress and areas needing improvement. Concurrently, students are encouraged to provide feedback to teachers. This bidirectional communication mechanism enhances the interaction between teachers and students, promoting continuous improvement in teaching methods and the learning process. Increased student teacher interaction has been observed in various student-centered, new teaching models that emphasize active student participation and collaborative student teacher interaction [43, 44].

Consistent with other studies, our research found that students’ satisfaction of teaching mode was significantly higher in the CDIO model group compared to the traditional control group [45]. In the CDIO model, the use of real-world cases for student analysis and learning serves to increase interest and satisfaction; CDIO emphasizes active student engagement in the learning process and self-resolution of practical problems, positioning students in a leading role within educational activities. This enhances their sense of participation, which is also a contributing factor to increased satisfaction.

Our study followed the CDIO process, guiding students to participate throughout. We investigated whether the CDIO model surpasses the traditional LBL method in knowledge retention after 24-week. Our findings suggest that after the implementation of the CDIO model, students’ scores in application abilities exceeded those achieved through traditional LBL method, while scores based on memory of theoretical knowledge showed no statistical difference in delayed tests. This indicates that the CDIO model is more beneficial for long-term improvement in application abilities in teaching students about arrhythmias.

## Conclusion

Our study pioneers the CDIO model’s application in arrhythmia courses for nursing students, enhancing their theoretical knowledge and application capability. This effective, innovative approach shows promise in clinical skills enhancement, particularly in arrhythmia identification and management. While further research is needed to address potential biases and explore applicability to broader groups, initial findings suggest the CDIO model significantly improves learning outcomes, satisfaction, and interest among nursing students, meriting further exploration and potential expansion to additional trainees.

### Study limitation

The study presented here encounters several main limitations. Firstly, the investigation was primarily focused on trainee nurses, which limits the generalizability of the findings. To validate the effectiveness of the proposed combined method, it’s imperative to conduct future studies with a more diverse participant pool, such as internal and surgical resident physicians, dentists, and public health service personnel, among others. Secondly, due to the limited sample size of this study, additional research with a larger cohort is essential to fully evaluate the impact of the method. Moreover, this study did not explore the longer-term retention and application of knowledge by the participants. Future research should include more time points, such as 36-week and 48-week post-class assessments, to investigate the durability of retained knowledge.

### Electronic supplementary material

Below is the link to the electronic supplementary material.

Supplementary Material 1

## Data Availability

Please contact the corresponding author for data availability.
