# Supplementary material for: Advancing arrhythmia education through the CDIO approach: a new paradigm in nursing student training
Source: BMC Nurs. 2024 Jun 25;23:427. doi: 10.1186/s12912-024-02118-1 (PMC11197327; doi:10.1186/s12912-024-02118-1)
Supplement: Supplementary file 1 — Supplementary Material 1 [file 12912_2024_2118_MOESM1_ESM.docx]

**Supplemental Material**

**Supplemental figure legends**

Figure S1. Examples of questions from the basic theoretical knowledge section.


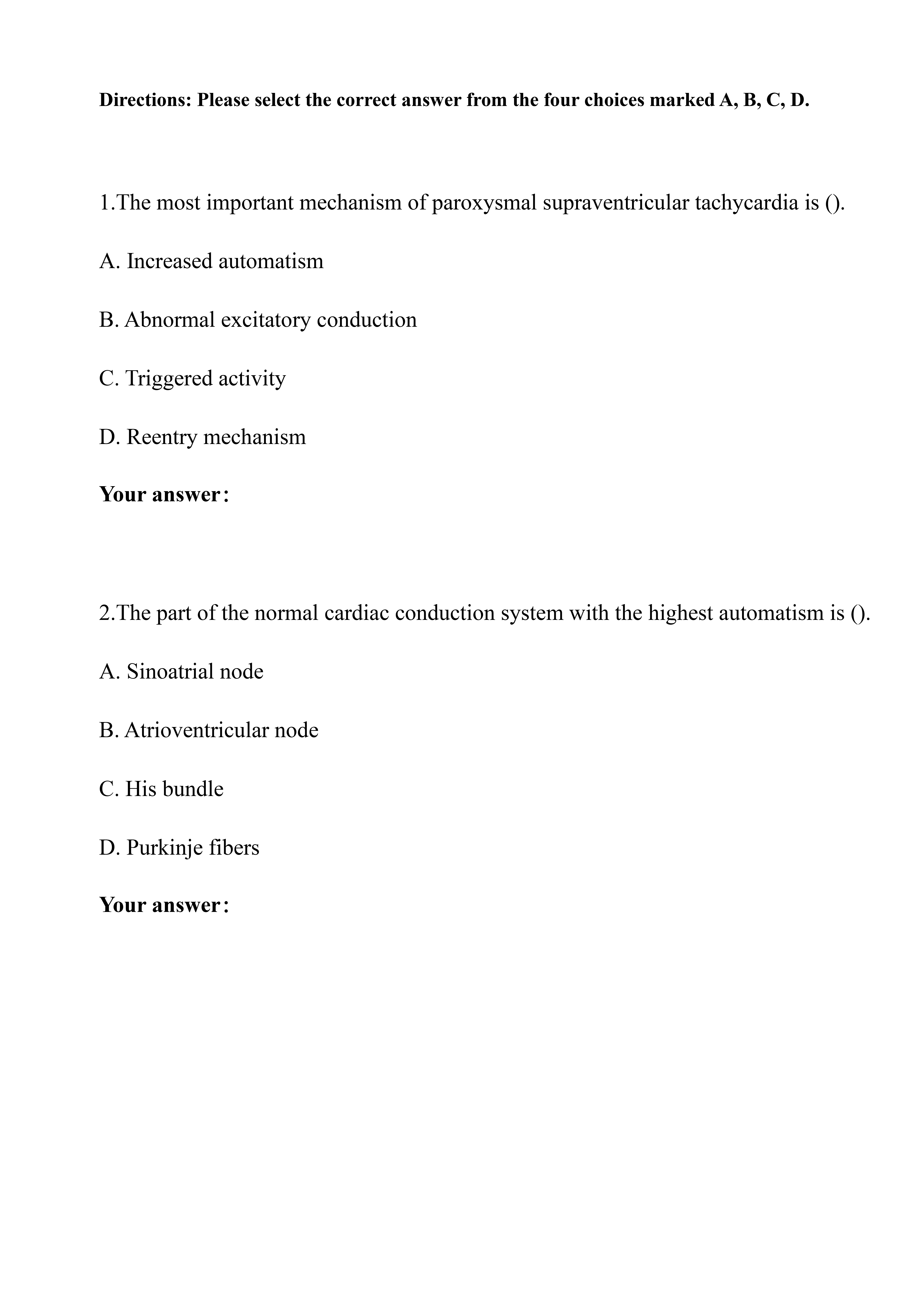


Figure S2. Examples of questions from the clinical case analysis section.


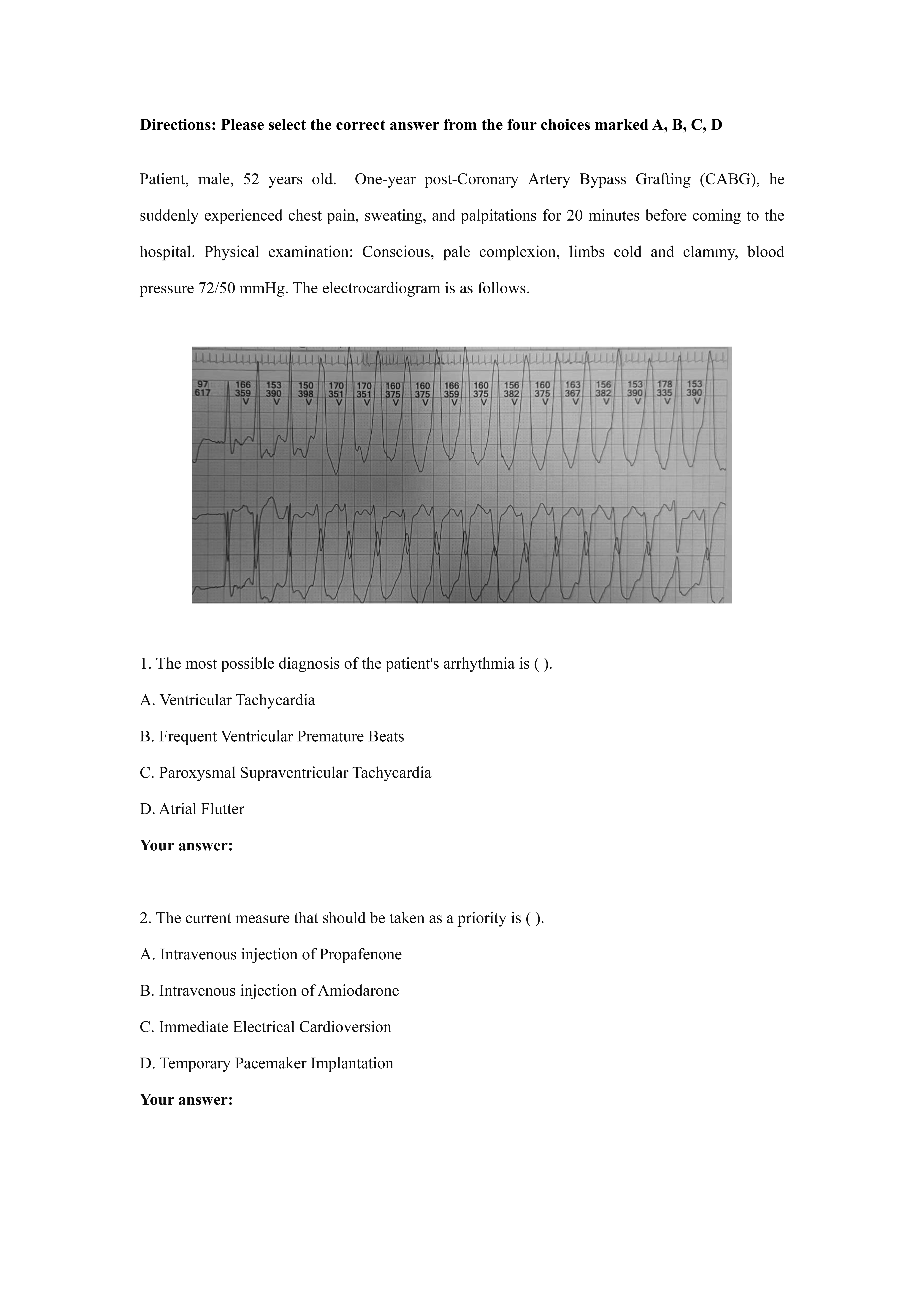


**Supplemental Table**

Table S1. Students’ attitudes towards the teaching modes

| Questions |
| --- |
| 1. This teaching mode increases your self-learning enthusiasm.   1 strongly disagreed, 2 disagreed, 3 neutral, 4 agreed, 5 strongly agreed   1. This teaching mode increases your study load.   1 strongly disagreed, 2 disagreed, 3 neutral, 4 agreed, 5 strongly agreed   1. This teaching mode has systematization of teaching content.   1 strongly disagreed, 2 disagreed, 3 neutral, 4 agreed, 5 strongly agreed   1. This teaching mode was helpful in understanding of teaching content.   1 strongly disagreed, 2 disagreed, 3 neutral, 4 agreed, 5 strongly agreed   1. This teaching mode has good student teacher interaction.   1 strongly disagreed, 2 disagreed, 3 neutral, 4 agreed, 5 strongly agreed   1. Are you satisfied with this teaching mode.   1 very dissatisfied, 2 dissatisfied, 3 neutral, 4 satisfied, 5 very satisfied   1. Are you satisfied with the teaching effect.   1 very dissatisfied, 2 dissatisfied, 3 neutral, 4 satisfied, 5 very satisfied   1. This teaching mode helps with the development of your self-confidence.   1 strongly disagreed, 2 disagreed, 3 neutral, 4 agreed, 5 strongly agreed   1. This teaching mode helps to foster team collaboration.   1 very dissatisfied, 2 dissatisfied, 3 neutral, 4 satisfied, 5 very satisfied   1. This teaching mode increases your interests in continuing to learn about Arrhythmia   1 strongly disagreed, 2 disagreed, 3 neutral, 4 agreed, 5 strongly agreed |

Table S2. Statistic and *p* value of arrhythmia test scores

|  |  |  | | ***F*** | ***p*** |
| --- | --- | --- | --- | --- | --- |
| **Total scores** | Main effect and Interaction effect | Time | | 420.770 | ＜0.001 |
|  |  | Group | | 36.098 | ＜0.001 |
|  |  | Time*Group | | 7.852 | 0.001 |
|  | Simple effect | Time | Pre course | 0.734 | 0.393 |
|  |  |  | 1w after course | 27.962 | ＜0.001 |
|  |  |  | 24w after course | 15.540 | ＜0.001 |
|  |  | Group | Exp | 283.159 | ＜0.001 |
|  |  |  | Con | 163.439 | ＜0.001 |
| **Theoretical scores** | Main effect and Interaction effect | Time | | 189.630 | ＜0.001 |
|  |  | Group | | 12.116 | 0.001 |
|  |  | Time*Group | | 4.083 | 0.018 |
|  | Simple effect | Time | Pre course | 0.011 | 0.917 |
|  |  |  | 1w after course | 16.182 | ＜0.001 |
|  |  |  | 24w after course | 3.694 | 0.057 |
|  |  | Group | Exp | 126.013 | ＜0.001 |
|  |  |  | Con | 69.933 | ＜0.001 |
| **Application scores** | Main effect and Interaction effect | Time | | 270.177 | ＜0.001 |
|  |  | Group | | 23.681 | ＜0.001 |
|  |  | Time*Group | | 4.557 | 0.012 |
|  | Simple effect | Time | Pre course | 1.194 | 0.169 |
|  |  |  | 1w after course | 15.714 | ＜0.001 |
|  |  |  | 24w after course | 10.140 | 0.002 |
|  |  | Group | Exp | 172.044 | ＜0.001 |
|  |  |  | Con | 102.690 | ＜0.001 |

Con: control group with the traditional LBL method. Exp: experimental group with the CDIO model.
